# Development of an In Vitro Assessment Method for Chemotherapy-Induced Peripheral Neuropathy (CIPN) by Integrating a Microphysiological System (MPS) with Morphological Deep Learning of Soma and Axonal Images

**DOI:** 10.3390/toxics11100848

**Published:** 2023-10-10

**Authors:** Kazuki Matsuda, Xiaobo Han, Naoki Matsuda, Makoto Yamanaka, Ikuro Suzuki

**Affiliations:** 1 Department of Electronics, Graduate School of Engineering, Tohoku Institute of Technology, 35-1 Yagiyama Kasumicho, Taihaku-ku, Sendai 982-8577, Japan; matsudak0108@gmail.com (K.M.); xiaobohan@tohtech.ac.jp (X.H.); na-matsuda@tohtech.ac.jp (N.M.); 2 Business Creation Division Organs on Chip Project, Usio Inc., 1-6-5 Marunouchi, Chiyoda-ku, Tokyo 100-8150, Japan; m.yamanaka@ushio.co.jp

**Keywords:** chemotherapy, paclitaxel, oxaliplatin, vincristine, bortezomib, suramin, peripheral nerve injuries, dorsal root ganglion, microphysiological system, deep learning

## Abstract

Several anticancer drugs used in cancer therapy induce chemotherapy-induced peripheral neuropathy (CIPN), leading to dose reduction or therapy cessation. Consequently, there is a demand for an in vitro assessment method to predict CIPN and mechanisms of action (MoA) in drug candidate compounds. In this study, a method assessing the toxic effects of anticancer drugs on soma and axons using deep learning image analysis is developed, culturing primary rat dorsal root ganglion neurons with a microphysiological system (MPS) that separates soma from neural processes and training two artificial intelligence (AI) models on soma and axonal area images. Exposing the control compound DMSO, negative compound sucrose, and known CIPN-causing drugs (paclitaxel, vincristine, oxaliplatin, suramin, bortezomib) for 24 h, results show the somatic area-learning AI detected significant cytotoxicity for paclitaxel (* *p* < 0.05) and oxaliplatin (* *p* < 0.05). In addition, axonal area-learning AI detected significant axonopathy with paclitaxel (* *p* < 0.05) and vincristine (* *p* < 0.05). Combining these models, we detected significant toxicity in all CIPN-causing drugs (** *p* < 0.01) and could classify anticancer drugs based on their different MoA on neurons, suggesting that the combination of MPS-based culture segregating soma and axonal areas and AI image analysis of each area provides an effective evaluation method to predict CIPN from low concentrations and infer the MoA.

## 1. Introduction

Several anticancer drugs used in cancer treatment induce chemotherapy-induced peripheral neuropathy (CIPN) as an adverse effect. CIPN manifests on the hands and feet, often in the “glove and stocking” distribution, with two types of sensory abnormalities. One is manifested as a prickling or burning sensation and the other as numbness and diminished touch sensation. These CIPN symptoms may be acute or chronic, with nearly 90% of patients developing at least one acute neuropathic symptom during the first treatment cycle [1]. The incidence of chronic CIPN tends to range widely from 13% to 70% [2,3,4] depending on the type and dose of chemotherapy, and it may persist for several years after treatment is discontinued. Common methods to assess CIPN severity are the National Cancer Institute—Common Toxicity Criteria (NCI-CTC) and the total neuropathy score (TNS) [5]. The severity and incidence of anticancer drugs known to cause CIPN (taxanes, vinca alkaloids, platinum compounds, proteasome inhibitors) varies depending on the administration period, administration schedule, administration method, and dose [5,6,7,8]. Depending on the severity of the impairment, limitations on the dosage of anticancer drugs or even discontinuation of treatment may be necessary. While therapeutic efficacy is a priority in anticancer drug development, the assessment of CIPN has become an important test parameter. Tests No. 424 and 426 in the Organization for Economic Cooperation and Development (OECD) guidelines for chemical testing recommend experiments using rats to obtain the information necessary to identify and confirm the neurotoxicity of chemicals [9,10]. However, animal experiments are problematic due to their high costs and lengthy durations. In recent years, the FDA Modernization Act 2.0 has led to an increased demand for in vitro new approach methodologies (NAMs) as alternatives to animal testing [11]. One example of in vitro NAMs is the Developmental Neurotoxicity In Vitro Battery proposed by OECD [12,13,14,15,16]. This battery consists of eight modules organized according to modeled neurodevelopmental processes (Proliferation, Apoptosis, Migration, Neuronal Differentiation, Neurite Outgrowth, Neurite Maturation and Synaptogenesis, Glial Differentiation, Neural Network Formation) and corresponding to 17 assays [13,14,15,16,17,18].

Over the years, various in vitro models for CIPN have been established, enhancing their applicability. However, the clinical symptoms of CIPN and the cells and molecular targets involved vary depending on the type of anticancer drug [19], leading to diverse evaluation methods. For instance, oxaliplatin, a platinum-based chemotherapy drug used for solid tumors, forms DNA adducts in cells and inhibits DNA replication [20]. Evaluation methods for oxaliplatin include measuring cell death using the terminal deoxynucleotidyl transferase dUTP nick-end labeling assay and evaluating changes in cell morphology and size distribution [21,22], mainly focusing on cellular effects. On the other hand, taxane-based chemotherapy drugs like paclitaxel and vinca-alkaloid-based drugs like vincristine cause excessive microtubule polymerization, inhibiting normal microtubule dynamics [20]. Evaluation methods for these drugs include morphological assessments quantifying neurite lengths using neuronal markers like Tuj-1 (βIII-tubulin) [23,24]. Paclitaxel has also been reported to exhibit cytotoxic effects on Schwann cells, which form myelin [25]. Coculturing rat dorsal root ganglion (DRG) neurons and Schwann cells enables the evaluation of myelin density through high-content imaging analysis of Myelin Basic Protein (MBP) markers [25].

In other words, CIPN affects soma, axons, and myelin, and the evaluation of candidate compounds for CIPN in vitro requires the comprehensive observation of changes in these components. As a method for comprehensive observation, machine-learning-based methods have been studied in recent years. Recent advancements have shown that convolutional neural networks (CNNs), a type of deep learning model, have superior accuracy in analyzing cell morphology images [26]. However, while these machine learning methodologies are adept at image analysis, their precision is contingent upon the caliber of the training dataset. Thus, during the assessment of effects on the soma, any image information unrelated to the soma acts as a source of noise. Similarly, when evaluating the impact on axons, information pertaining to the soma can introduce noise, subsequently diminishing predictive accuracy.

Therefore, this study aimed to develop a method for predicting the toxic effects of anticancer drugs and whether they impact soma or axons using deep learning on images. To predict target sites, a microphysiological system (MPS) was employed to separate soma and axonal areas within the same sample and acquire distinct images [27]. By training two artificial intelligence (AI) models on the images of these distinct areas, an attempt was made to classify the mechanisms of anticancer drugs that affect different areas in neuronal cells.

## 2. Materials and Methods

### 2.1. Device Fabrication

**Figure 1:**
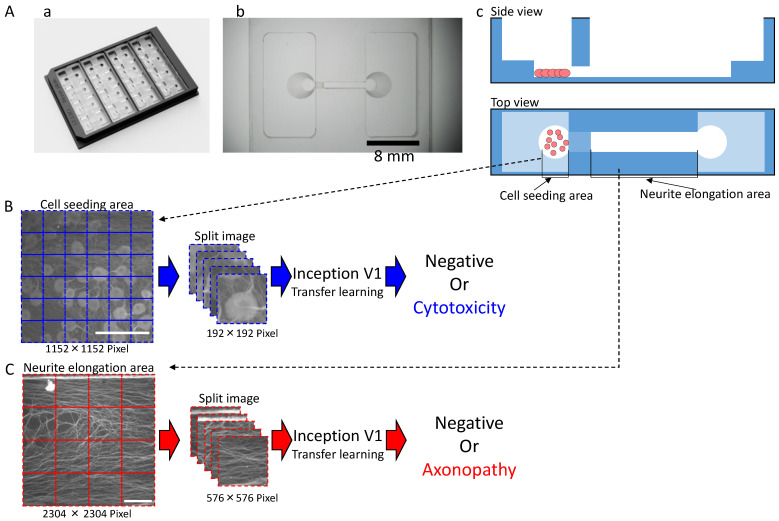
Shape of the microphysiological system (MPS) and the process for dataset processing and artificial intelligence (AI) analysis for deep learning: (**A**) MPS with (**a**) an overview; (**b**) Magnified view of a single channel. Scale bar = 8 mm; (**c**) Schematic of cultivation. (**B**) Dataset processing and AI analysis scheme for the cell seeding area. Scale bar = 200 μm. (**C**) Dataset processing and AI analysis scheme for the neuronal protrusion extension area. Scale bar = 200 μm.

The MPS (Ushio Inc., Tokyo, Japan) was constructed as previously described [28]. In brief, vacuum ultraviolet (VUV) photobonding from an excimer light at a 172 nm wavelength was used to generate functional groups (i.e., hydroxy and carboxyl groups) for directly combining two COP material layers under heat treatment. Individual cell culture channels are aligned in scaled platform for high through output (Figure 1A). The narrow middle slot of the cell culture channel is 1000 µm in width, 165 µm in length, and 40 µm in height, with an open-top channel (1000 µm in width and 6 mm in length) and two circular holes (2 mm in diameter) at both ends that open into a medium rectangular reservoir (15 mm in width, 8 mm in length, and 5 mm in height). The maximum volume contained in each channel is 1 mL. COP material (Zeonex 690R, Zeon, Tokyo, Japan) was individually injected into the two molds. The components were irradiated with VUV from an excimer lamp (172 nm; Ushio Inc., Tokyo, Japan) at 25 °C after removing the structured COP components from the molds. The component surfaces were assembled using a heat press at <132 °C. Finally, ethylene oxide gas (Japan Gas Co. Ltd., Kanagawa, Japan) was used to decontaminate the device.

### 2.2. Cell Culture

Before cell seeding, the microfluidic channel was coated with 0.02% Poly-L-lysine (P4707, Sigma–Aldrich, Burlington, NJ, USA) overnight at 4 °C. After washing with phosphate-buffered saline (PBS), the device was coated with 2.5 µg/mL laminin 511 (381-07363, Wako, Osaka, Japan) for 30 min at 37 °C. Primary rodent DRG neurons were harvested and cultured as described previously [29]. All procedures were performed according to the Guide for the Care and Use of Laboratory Animals published by the US National Institutes of Health [30] and were approved by the Tohoku Institute of Technology Animal Care and User Committee. Briefly, DRG neurons were collected from embryos of one-time pregnant (E14) Sprague Dawley rat (total ~10 embryos). Firstly, the rats were asphyxiated with isoflurane and embryos were recollected. Then, spinal cords with DRGs were carefully isolated and removed from embryos. After plucking off DRGs from the spinal cords, the sensory neurons were dissociated by incubation for 30 min with 0.25% Trypsin at 37 °C. After cell counting, approximately 5.0 × 10^4^ dissociated DRG neurons in 25 µL Neurobasal neuronal medium (with B-27 supply, Gibco, Billings, USA) were seeded directly into the cell seeding area of each channel. After 1 h, another 600 µL of Neurobasal neuronal medium was applied to the whole channel. The next day, the medium was replaced with 600 µL of Neurobasal neuronal medium containing 1 µM ara-C kept for 3 days to suppress the proliferation of glial cells. Afterwards, the medium was changed back to 600 µL Neurobasal neuronal medium, and half the volume of the medium was replaced twice per week. After 2 weeks (14 days) in culture, three typical anticancer drugs were administered to the cultures at two different concentrations each: paclitaxel at 0.1 µM and 1 µM, vincristine at 0.003 µM and 0.03 µM, and oxaliplatin at 10 µM and 100 µM. As validation compounds, bortezomib at 0.01 µM, a proteasome inhibitor, and suramin at 10 µM and 100 µM, antiparasitic drugs that have antineoplastic effects and cause myelin damage, were administered. DMSO (0.1%) and sucrose 10 µM were added as two negative control drugs to the cultures. Bortezomib at 0.01 µM was added as a testing drug to the cultures. The drug exposure lasted for 24 h at 37 °C.

### 2.3. Immunocytochemistry

After drug exposure, the sample cultures were fixed with 4% paraformaldehyde in PBS (at 4 °C) for 10 min. Fixed cells were then incubated with 0.2% Triton-X-100 in PBS for 5 min, then with preblock buffer (0.05% Triton-X and 5% FBS in PBS) at 4 °C for 1 h, and finally with preblock buffer containing a specific primary antibody, mouse anti-β-tubulin III (1:1000, T8578, Sigma–Aldrich), at 4 °C overnight. The sample cultures were then incubated with a secondary antibody, antimouse 488 Alexa Fluor (1:1000 in preblock buffer, ab150113, Abcam, Tokyo, Japan), for 1 h at room temperature. Stained cultures were washed twice with preblock buffer and rinsed twice with PBS. Local images of cell seeding area and neurite elongation area were captured by a confocal microscope (Eclipse Ti2-U, Nikon, Tokyo, Japan). ImageJ software (NIH) was used to adjust image intensity.

### 2.4. Deep Learning for Cell Seeding Area Prediction and Neurite Elongation Area Prediction

A local image (2304 × 2304 pixel) of the soma area was divided into 4 segments (1152 × 1152 pixel) and used as the dataset image for the cytotoxicity prediction AI analysis. The dataset for cytotoxicity prediction AI consisted of DMSO (*n* = 32), sucrose at 10 µM (*n* = 36), paclitaxel at 0.1 µM (*n* = 38), paclitaxel at 1 µM (*n* = 10), vincristine at 0.003 µM (*n* = 13), vincristine at 0.03 µM (*n* = 24), oxaliplatin at 10 µM (*n* = 20), oxaliplatin at 100 µM (*n* = 16), suramin at 10 µM (*n* = 20), suramin at 100 µM (*n* = 16), and bortezomib at 0.01 µM (*n* = 8). The 1152 × 1152-pixel image was segmented into 192 × 192-pixel images. The segmented 192 × 192-pixel images were used for training and prediction. The percentage of segmented images that were determined to be positive out of the 1152 × 1152-pixel images was calculated as the toxicity probability.

A local image (2304 × 2304 pixel) of the axonal area was used as the dataset image for AI analysis. The dataset for AI predicting axonopathy included DMSO (*n* = 16), sucrose at 10 µM (*n* = 10), paclitaxel at 0.1 µM (*n* = 10), paclitaxel at 1 µM (*n* = 10), vincristine at 0.003 µM (*n* = 13), vincristine at 0.03 µM (*n* = 15), oxaliplatin at 10 µM (*n* = 16), oxaliplatin at 100 µM (*n* = 15), suramin at 10 µM (*n* = 10), suramin at 100 µM (*n* = 13), and bortezomib at 0.01 µM (*n* = 6).

The 2304 × 2304-pixel image was segmented into 576 × 576-pixel images. The segmented 576 × 576-pixel image was used for training and prediction. The percentage of segmented images in the 2304 × 2304-pixel images that were determined to be positive was calculated as the toxicity probability.

### 2.5. Statistical Analysis

A one-way analysis of variance (ANOVA) followed by a Dunnett test was used to calculate significant differences between DMSO and the compounds in the predictive accuracy of AI. For the test of two-dimensional plots to classify the mechanisms of action (MoA), a one-way multivariate analysis of variance (MANOVA) was used to calculate significant differences between the compounds and between each concentration.

### 2.6. Grad-CAM

Gradient-weighted Class Activation Mapping (Grad-CAM) was used to visualize areas of interest during toxicity determination; Grad-CAM incorporated class-specific gradient information into the final CNN’s convolution layer to visualize important image areas [31].

## 3. Results

### 3.1. Morphological Changes in DRG Neurons by CIPN-Inducing Compounds

**Figure 2:**
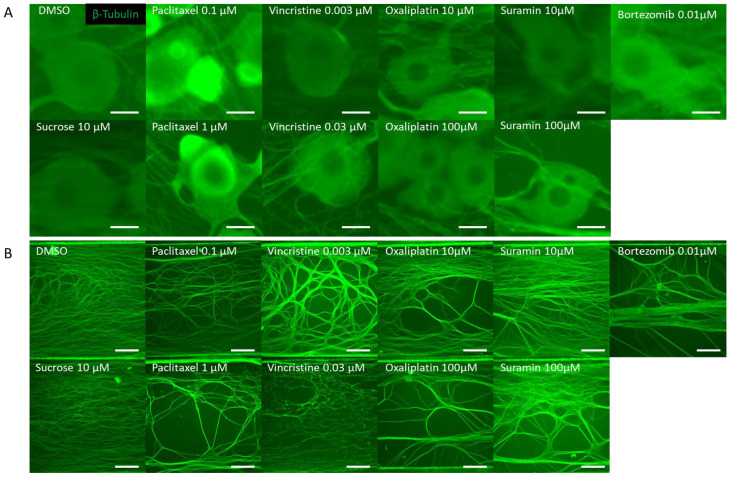
Representative β-Tubulin immunofluorescence images in the microfluidic device after drug administration. To verify the response to the compounds, rat dorsal root ganglion (DRG) neurons were cultured on the MPS device, exposed to the compound on day 14, and immunostaining images were taken 24 h later. The compounds used included DMSO as a vehicle, sucrose as a negative compound, paclitaxel and vincristine as anticancer drugs that cause axonal damage, and oxaliplatin, which induces somatic cell damage. Additionally, bortezomib, a proteasome inhibitor reported to cause chemotherapy-induced peripheral neuropathy (CIPN), and suramin, an antiparasitic drug with antitumor effects known to cause myelin damage, were selected as test compounds: (**A**) Representative local immunofluorescence images of soma. From left: DMSO 0.1% and sucrose at 10 µM. Paclitaxel at 0.1 µM and paclitaxel at 1 µM. Vincristine at 0.003 µM and vincristine at 0.03 µM. Oxaliplatin at 10 µM and oxaliplatin at 100 µM. Suramin at 10 µM and suramin at 100 µM. Bortezomib at 0.01 µM. Scale bar = 20 μm. (**B**) Representative local immunofluorescence images of axons. From left: DMSO 0.1% and sucrose at 10 µM. Paclitaxel at 0.1 µM and paclitaxel at 1 µM. Vincristine at 0.003 µM and vincristine at 0.03 µM. Oxaliplatin at 10 µM and oxaliplatin at 100 µM. Suramin at 10 µM and suramin at 100 µM. Bortezomib at 0.01 µM. Scale bar = 200 μm.

DRG neurons were cultured on MPS devices. On the 14th day, they were exposed to various anticancer drugs and, subsequently, 24 h post exposure, immunostained images were captured. Tested drugs included paclitaxel and vincristine, known inducers of axonal damage, as well as oxaliplatin, which causes soma damage. Sucrose and DMSO were utilized as negative controls. For validation, bortezomib, a proteasome inhibitor associated with CIPN, and suramin, an antiparasitic drug with antineoplastic effects but known to damage myelin, were chosen. A distinctive feature of this MPS is its ability to culture the soma and axonal areas separately. Notably, the axonal area is openly structured, ensuring no hindrance to projection elongation (Figure 1A). Following MPS-based culture, distinct images of the soma and axons were captured post immunostaining. Morphological alterations within both soma and axonal domains in response to the compounds were evaluated (Figure 2). Figure 2A shows the representative immunofluorescence images of soma post compound exposure. Following a 24 h exposure, no noticeable morphological alterations were seen in the soma treated with DMSO, sucrose, or vincristine. However, oxaliplatin and suramin treatments led to observable soma reduction and contour deformation. Moreover, both low and high concentrations of paclitaxel induced soma shrinkage, contour irregularities, and changes in luminance values. Figure 2B shows representative immunofluorescence images of axons post compound exposure. Both DMSO and sucrose had no effect on axonal morphology. At low concentrations of paclitaxel, oxaliplatin, and suramin, some axons exhibited aggregation. At high concentrations, suramin and vincristine induced significant axonal aggregation and transection.

### 3.2. Toxicity Prediction Based on Compound-Induced Morphological Changes Using Two AI Models, Soma and Axonal Areas

In order to have toxicity predicted from the acquired soma and axon images, two different AIs were created to predict the toxicity of each (Figure 1B,C). The cell and axon image datasets used to create each toxicity prediction AI are shown in Table 1 and Table 2. Drugs used for training data for the toxicity prediction AI in the soma area were DMSO, sucrose, and oxaliplatin at 10 µM and 100 µM, and the number of samples ranged from *n* = 8 to *n* = 24, depending on the compound. The drugs used in the validation data were DMSO, sucrose, oxaliplatin at 10 μM and 100 μM, paclitaxel at 0.1 μM and 1 μM, vincristine at 0.003 μM and 0.03 μM, suramin at 10 μM and 100 μM, and bortezomib at 0.01 μM, and the number of samples ranged from *n* = 8 to *n* = 38, depending on the compound.

The drugs used for the training data for the toxicity prediction AI in the axonal area were DMSO, sucrose, and vincristine at 0.003 μM and 0.03 μM, and the number of samples ranged from *n* = 7 to *n* = 10, depending on the compound. The drugs used in the validation data were DMSO, sucrose, vincristine at 0.003 μM and 0.03 μM, paclitaxel at 0.1 μM and 1 μM, oxaliplatin at 10 μM and 100 μM, suramin at 10 μM and 100 μM, and bortezomib at 0.01 μM, and the number of samples ranged from *n* = 3 to *n* = 16, depending on the compound.

**Figure 3:**
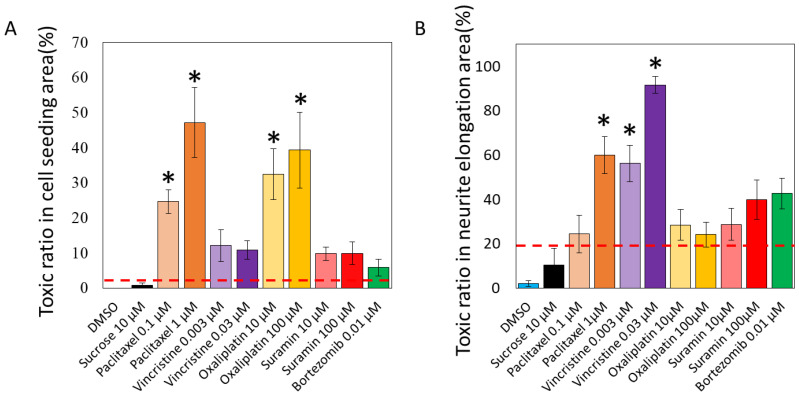
Predicted toxicity positive rates of various compounds by AI analysis. Two AI models were developed to detect the toxicity in the acquired cellular and axonal images. The ratio of segmented images identified as positive within a single image was calculated as the toxicity probability: (**A**) The predictive model for cytotoxicity revealed that paclitaxel was associated with 24.6 ± 3.3% (0.1 μM), 47.1 ± 10.0% (1 μM), vincristine with 12.1 ± 4.5% (0.003 μM), 10.9 ± 2.6% (0.03 μM), oxaliplatin with 32.5 ± 7.2% (10 μM), 39.3 ± 10.8% (100 μM), suramin with 9.8 ± 1.9% (10 μM), 9.9 ± 3.2% (100 μM), and bortezomib with 5.8 ± 2.4% (0.01 μM). The toxicity probabilities for DMSO and sucrose were 0.0 ± 0.0% and 0.7 ± 0.7%, respectively, indicating a low toxicity positivity rate. The mean of the toxicity positivity rates of these negative compounds plus twice the standard deviation was set as the toxicity prediction line (3.3%). One-way analysis of variance (ANOVA) was used for statistical analysis, followed by Dunnett’s test. * *p* < 0.05 vs. DMSO. (**B**) The predictive model for axonopathy revealed that paclitaxel was associated with 24.3 ± 8.4% (0.1 μM), 60.0 ± 8.4% (1 μM), vincristine with 56.3 ± 8.1% (0.003 μM), 91.7 ± 3.8% (0.03 μM), oxaliplatin with 28.5 ± 6.8% (10 μM), 24.2 ± 5.6% (100 μM), suramin with 28.8 ± 7.2% (10 μM), 39.9 ± 8.8% (100 μM), and bortezomib with 42.7 ± 3.0% (0.01 μM). The toxicity probabilities for DMSO and sucrose were predicted to be 2.1 ± 1.3% and 10.4 ± 7.5%, respectively, which are low toxicity positivity rates. The mean of the toxicity positivity rates for these compounds plus twice the standard deviation was set as the predicted toxicity line (21.1%). One-way ANOVA was used for statistical analysis, followed by Dunnett’s test. * *p* < 0.05 vs. DMSO.

The results of the toxicity prediction of the unlearned soma images are shown in Figure 3A. The percentage of segmented 192 × 192-pixel images in a single 1152 × 1152-pixel image that tested positive was calculated as the toxicity probability (%).

The predictive model for cytotoxicity revealed that paclitaxel was associated with 24.6 ± 3.3% (0.1 μM), 47.1 ± 10.0% (1 μM), vincristine with 12.1 ± 4.5% (0.003 μM), 10.9 ± 2.6% (0.03 μM), oxaliplatin with 32.5 ± 7.2% (10 μM), 39.3 ± 10.8% (100 μM), suramin with 9.8 ± 1.9% (10 μM), 9.9 ± 3.2% (100 μM), and bortezomib with 5.8 ± 2.4% (0.01 μM). The toxicity probabilities for DMSO and sucrose were 0.0 ± 0.0% and 0.7 ± 0.7%, respectively, indicating a low toxicity positivity rate. The mean of the toxicity positivity rates of these negative compounds plus twice the standard deviation was set as the toxicity prediction line (3.3%). As a result, all anticancer drugs and suramin exceeded the positive line, even at low concentrations. In particular, oxaliplatin, which causes soma damage, had a significantly higher toxicity positivity rate than DMSO at all concentrations, and significant toxicity to the soma was detected. Paclitaxel, which causes axonal damage, also showed a significantly higher rate of positive toxicity compared with DMSO at all concentrations, and significant toxicity to the soma was detected. Vincristine, which causes axonal damage, was detected to be toxic to the soma but not significantly so compared with DMSO. The validated compounds bortezomib and suramin were detected to be toxic but not significantly so.

The results of toxicity prediction for unlearned axonal images are shown in Figure 3B. The percentage of segmented 576 × 576-pixel images in a single 2304 × 2304-pixel image that tested positive was calculated as the toxicity probability (%). The predictive model for axonopathy revealed that paclitaxel was associated with 24.3 ± 8.4% (0.1 μM), 60.0 ± 8.4% (1 μM), vincristine with 56.3 ± 8.1% (0.003 μM), 91.7 ± 3.8% (0.03 μM), oxaliplatin with 28.5 ± 6.8% (10 μM), 24.2 ± 5.6% (100 μM), suramin with 28.8 ± 7.2% (10 μM), 39.9 ± 8.8% (100 μM), and bortezomib with 42.7 ± 3.0% (0.01 μM). The toxicity probabilities for DMSO and sucrose were predicted to be 2.1 ± 1.3% and 10.4 ± 7.5%, respectively, which are low toxicity positivity rates. The mean of the toxicity positivity rates for these compounds plus twice the standard deviation was set as the predicted toxicity line (21.1%). As a result, all anticancer drugs and suramin exceeded the positive line, even at low concentrations. A significantly higher rate of positive toxicity was detected for high concentrations of paclitaxel, which causes axonal damage, and for vincristine in particular compared with DMSO, with pronounced toxicity to axons. Oxaliplatin, which causes soma damage, was detected to be toxic to axons but not significantly so compared with DMSO. The validated compounds bortezomib and suramin were detected to be toxic but not significantly so.

### 3.3. Classification of MoA Based on Toxicity Prediction Results from Two AI Models

**Figure 4:**
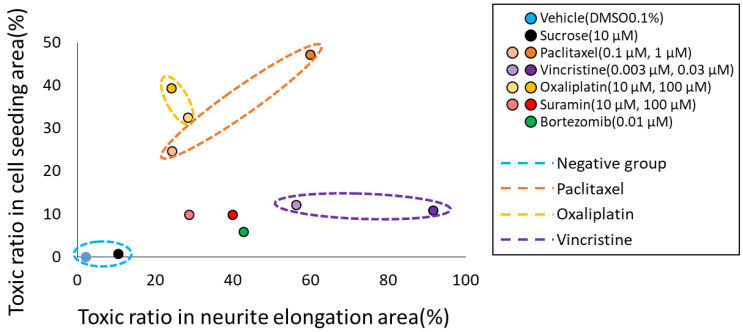
Classification of the mechanisms of action (MoA) of each compound based on the prediction results of two types of AI. To classify the MoA of anticancer drugs on neurons, we utilized the toxicity probability results from two distinct AI models. The toxicity probability in the soma area was taken as the vertical axis and in the axonal area as the horizontal axis. The graphical representation indicated that both DMSO and sucrose clustered near the origin. Meanwhile, oxaliplatin exhibited a predominant shift in the *y*-axis direction. In contrast, paclitaxel shifted in the upper-right quadrant, indicating a simultaneous increase in both axonopathy and cytotoxicity. Vincristine primarily moved in the *x*-axis direction, indicating its pronounced effect on axonal areas. The validation compound, suramin, showcased a dose-dependent shift along the *x*-axis. Another validation compound, bortezomib, positioned itself close to suramin at the 100 μM point.

To classify the MoA of anticancer drugs on neurons, we utilized the toxicity probability results from two distinct AI models. Specifically, the toxicity probability in the soma area was taken as the vertical axis and in the axonal area as the horizontal axis. The outcomes for each compound were plotted to examine their distributions (Figure 4). The graphical representation indicated that both DMSO and sucrose clustered near the origin. Meanwhile, oxaliplatin exhibited a predominant shift in the *y*-axis direction. In contrast, paclitaxel shifted in the upper-right quadrant, indicating a simultaneous increase in both axonopathy and cytotoxicity. Vincristine primarily moved in the *x*-axis direction, indicating its pronounced effect on axonal areas. The validation compound, suramin, showcased a dose-dependent shift along the *x*-axis. Another validation compound, bortezomib, positioned itself close to suramin at the 100 μM point. To further elucidate the separations among the compounds, a one-way MANOVA test was employed. The outcomes revealed significant differences among nearly all compounds. However, only suramin at 100 µM and bortezomib at 0.01 µM did not manifest significant differentiation (Table 3). This analysis suggests that the predictions derived from the two AI models hold potential in classifying anticancer drugs based on their specific mechanism, be it soma damage or axonal damage.

## 4. Discussion

**Figure 5:**
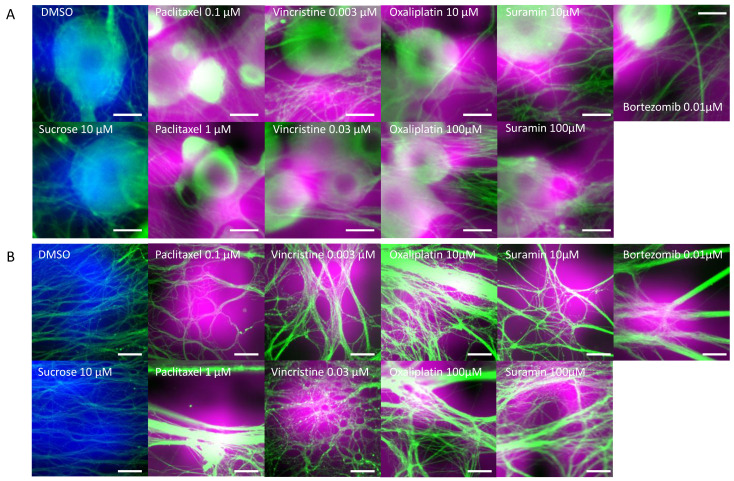
Areas of interest during AI toxicity determination. The areas that the AI focused on during toxicity determination were visualized using GradCAM: (**A**) Cellular areas on which AI focused during cellular toxicity determination. Areas colored in blue represent the areas focused on by the AI when cellular structures treated with DMSO and sucrose were determined to be negative. The areas colored in magenta represent the areas the AI focused on when cellular structures were determined to be damaged. Scale bar = 20 μm. (**B**) Axonal areas on which AI focused during axonopathy determination. Areas colored in blue represent the areas focused on by the AI when axons treated with DMSO and sucrose were determined to be negative. The areas colored in magenta represent the areas the AI focused on when axons were determined to be damaged. Scale bar = 50 μm.

In this study, we employed AI to determine toxicity from images of the soma and axonal areas. To ascertain if the AI could identify morphological abnormalities in these areas during toxicity determination, we visualized the areas of focus using Grad-CAM (Figure 5). Figure 5A displays the areas of the soma that the AI focused on during toxicity determination. The areas colored in blue indicate the areas the AI focused on when the soma treated with DMSO and sucrose was determined as negative, and the magenta-colored areas signify areas where the AI detected potential damage to the soma. Observations reveal that the AI typically focused on the transition from the center to the periphery of the soma when making a negative determination. In contrast, when detecting damage, the AI seemed to concentrate on the periphery of the soma and luminescent spots, suggesting that it considers changes in the soma’s shape and luminescence. Considering paclitaxel induces cell death [32], leading to mitochondrial and lysosomal release exhibiting autofluorescence [33], the AI’s damage determination based on luminescent spots appears valid. Furthermore, for compounds like oxaliplatin, vincristine, suramin, and bortezomib, the AI’s focus on the periphery and surroundings of the soma suggests it is detecting changes in the morphology and size of the soma, with such morphological changes being attributed to apoptosis induced by these compounds [23,29,34], further affirming the AI’s damage determination.

Next, Figure 5B depicts the axonal areas the AI focused on during axonopathy determination. The blue areas show areas the AI focused on when axons treated with DMSO and sucrose were determined as negative, while the magenta-colored areas indicate the axonal areas where the AI detected potential damage. Observations suggest that the AI focused broadly on the axons when making negative determinations, but for compounds like 0.1 μM paclitaxel and 10 μM suramin, it paid attention to axonal aggregation and the resultant cavities. Similarly, with 1 μM paclitaxel, 0.003 μM vincristine, oxaliplatin, and 100 μM suramin, the AI seemed to highlight areas with pronounced axonal aggregation, while at 0.03 μM vincristine, it focused on the evident axonal damage, aligning with reports of vincristine causing axonal degeneration, including fragmentation [35,36]. The axonal aggregation observed in all compounds, except for 0.03 μM vincristine, is believed to result from increased matrix metalloproteinase (MMP) expression due to compound exposure, leading to the degradation of the extracellular matrix (ECM) that binds axons to the device [37,38], causing damaged axons to migrate and aggregate. To conclusively identify this aggregation mechanism, future studies should measure MMP expression levels and evaluate the ECM.

To assess the accuracy of the AI’s toxicity prediction, a comparison was made with previous reports (Table 4). The cytotoxicity of paclitaxel as determined by AI showed significant differences from DMSO at concentrations of 0.1 μM and 1 μM, exhibiting pronounced toxicity (Figure 3A). This aligns with the report that paclitaxel causes cell death at 0.1 μM and shows peak cytotoxicity at 1 μM [32]. Axonopathy was detected beyond the positive line at 0.1 μM and showed significant difference from DMSO at 1 μM (Figure 3B). This is consistent with the report indicating reduced neurite length and toxicity from 0.01 μM [32]. The cytotoxicity of oxaliplatin determined by AI showed pronounced toxicity at 10 μM and 100 μM when compared with DMSO (Figure 3A). This is consistent with the report that cell death occurs at 10 μM and pronounced cytotoxicity is observed at 33.2 μM [29]. Axonopathy exceeded the positive line at concentrations of 10 μM and 100 μM (Figure 3B), consistent with the observation of reduced neurite length starting from 3.3 μM [29]. The AI-determined cytotoxicity for vincristine was detected beyond the positive line at 0.003 μM and 0.03 μM (Figure 3A). This result contradicts the report that 24 h exposure to vincristine does not induce cell death [23]. However, 72 h after drug removal, cell death progressed, beginning from 0.001 μM, with significant cell death observed at 0.1 μM [23]. Thus, the detected cytotoxicity, based on the positive line, may reflect early stages of minor cellular changes. Additionally, pronounced axonopathy was detected at 0.003 μM and 0.03 μM compared with DMSO (Figure 3B), which aligns with the observation of reduced axonal area from 0.001 μM [29]. The AI’s assessment of suramin cytotoxicity indicated a toxicity beyond the positive line at 10 μM and 100 μM (Figure 3A). This contradicts the report that suramin exposure of 300 μM for 48 h induces cell death and 100 μM induces cell death after 8 days [34]. The detected cytotoxicity might represent early stages of minor cellular changes, suggesting the potential to detect toxicity at lower concentrations and earlier exposure times. Axonopathy was also observed beyond the positive line at 10 μM and 100 μM (Figure 3B), contrasting with the report of reduced neurite length after 48 h exposure to suramin concentrations greater than 200 μM [39]. This suggests that, like cytotoxicity, axonopathy can be detected at lower concentrations and at an early stage after exposure. The AI assessment of bortezomib’s cytotoxicity indicated a toxicity beyond the positive line at 0.01 μM (Figure 3A). This result contradicts the report that no cell death was induced by a 24 h exposure [23]. However, it has been reported that cell death progresses 72 h after drug removal, and significant cell death occurs at 0.012 μM [23]. Additionally, axonopathy was detected beyond the positive line at 0.01 μM (Figure 3B). After a 24 h exposure to 1 μM of bortezomib, there was a significant decrease in neuronal protrusions, and after 72 h, a significant decrease in neuronal protrusions was observed starting from 0.03 μM, reported in [23]. From this, it is suggested that even for bortezomib, it is possible to detect both cellular cytotoxicity and axonopathy at earlier stages post exposure and at lower concentrations.

In summary, compounds showing noticeable differences from DMSO seemingly capture their toxicity accurately. The utility of the positive line to spot previously unobserved low-concentration toxicity and slow-reacting compounds hints at its potential for early detection. Detecting toxicity 24 h post exposure is likely attributed to AI’s enhanced imaging diagnostic capacities, promising quicker and more reliable evaluations. However, subsequent studies expanding the spectrum of nontoxic compounds and delving deeper into the validity of the toxicity line are essential.

In this study, we attempted to classify the MoA of each compound by combining two AI models that detected toxicity in each area. By integrating the two AI models, we were able to classify the mode of action for oxaliplatin, paclitaxel, and vincristine, capturing dose-dependent variations. Moreover, some compounds that did not show significant differences when analyzed by individual AIs displayed significance when assessed by the combined AI approach (Table 3). This integration allowed for more accurate detection of toxicity, even at lower concentrations. Notably, suramin and bortezomib, which did not show significant differences when evaluated by each AI separately, exhibited significant differences upon combined analysis. Furthermore, they could be distinguished from oxaliplatin, paclitaxel, and vincristine. However, one limitation was our inability to differentiate between suramin and bortezomib. This is believed to be due to our evaluation system not assessing suramin’s myelin damage mechanism and bortezomib’s proteasome functionality. In the future, constructing similar image-based AI evaluation systems for myelin damage and proteasome function beyond soma and axons might enhance drug classification accuracy and broaden the scope.

Importantly, our assessment method has validity for in vitro to in vivo extrapolation (IVIVE). Dose limitation is established in the clinical administration of anticancer drugs, and in this result, toxicity could be detected in vitro at a value close to dose limitation. As an example, it has been reported that paclitaxel infusion causes CIPN, and the rate of treatment interruption increases in a dose-dependent manner with blood concentration [40]. This report is consistent with the detection of dose-dependent toxicity in our form of evaluation method, which can be used to estimate the dose limitation at which treatment is discontinued in the preclinical phase and may be used to determine the dose of administration that prioritizes drug efficacy while reducing CIPN symptoms. In addition, in recent years, research has been conducted on how to detect demyelination and degeneration of axons using magnetic resonance imaging (MRI) instead of biomarkers [41]. Correlating the results of the present evaluation method with this MRI-based method may further prevent the risk of developing CIPN in the preclinical stage.

Although our results have a few limitations that warrant consideration, including the evaluation of myelin, in this study, we evaluated representative anticancer drugs and antitumor drugs (paclitaxel, oxaliplatin, vincristine, bortezomib, suramin). However, to allow for a broader evaluation, anticancer and gene therapy drugs with different MoA must also be considered, and to better ensure the efficacy of IVIVE research in humans, the effect of changes in blood concentrations due to differences in the metabolisms of these drugs must also be examined in detail. Moreover, since each drug has a different metabolic time scale [40,42], an approach to a variety of time scales is essential for each one’s evaluation. Therefore, a future challenge is to evaluate multiple drugs on different time scales.

The combination of the MPS-based evaluation system developed in this study, which separates the soma and axonal areas, and an AI image analysis of each area is expected to provide clues for estimating the MoA of unknown compounds. Furthermore, since the degree of impairment can also be evaluated, it is expected to be possible to rank the concentration-dependent toxicity and risk of drug candidate compounds. In other words, the combination of the MPS-based culture of the soma and axonal areas separately and AI image analysis of each area is an effective evaluation method to predict CIPN.

## 5. Conclusions

In this study, utilizing the MPS, we developed two AI models from cellular body area images and oriented axonal area images. These AIs were capable of detecting the peripheral neurotoxicity of compounds at low concentrations and within 24 h post exposure. By integrating the results from both AIs, it became feasible to predict the MoA of compounds that induce CIPN. The combined approach of MPS and AI developed in this research suggests an effective evaluation method for predicting both the potential CIPN and the MoA of pharmaceutical candidate compounds.

## Figures and Tables

**Table 1:** Details of the learning dataset and test dataset when creating the soma area AI.

	Training Dataset		Testing Dataset	
Compounds	Concentration (µM)	*n* (Images)	Concentration (µM)	*n* (Images)
DMSO	0.10%	24	0.10%	8
Sucrose	10	24	10	12
Oxaliplatin	10	12	10	8
	100	8	100	8
Paclitaxel	-	-	0.1	38
	-	-	1	10
Vincristine	-	-	0.003	13
	-	-	0.03	24
Suramin	-	-	10	20
	-	-	100	16
Bortezomib	-	-	0.01	8

**Table 2:** Details of the learning dataset and test dataset when creating the axonal area AI.

	Training Dataset		Testing Dataset	
Compounds	Concentration (µM)	*n* (Images)	Concentration (µM)	*n* (Images)
DMSO	0.10%	10	0.10%	6
Sucrose	10	7	10	3
Vincristine	0.003	8	0.003	5
	0.03	9	0.03	6
Paclitaxel	-	-	0.1	10
	-	-	1	10
Oxaliplatin	-	-	10	16
	-	-	100	15
Suramin	-	-	10	10
	-	-	100	13
Bortezomib	-	-	0.01	6

**Table 3:** One-way multivariate analysis of variance results between each compound.

Compounds	Concentration (µM)	vs. DMSO	vs. Sucrose	vs. Paclitaxel		vs. Vincristine		vs. Oxaliplatin		vs. Suramin		vs. Bortezomib
		0.10%	10	0.1	1	0.003	0.03	10	100	10	100	0.01
DMSO	0.10%	―	** *p* = 2.66 × 10^−6^	** *p* = 1.48 × 10^−21^	** *p* = 4.89 × 10^−40^	** *p* = 7.18 × 10^−43^	** *p* = 1.61 × 10^−136^	** *p* = 3.77 × 10^−29^	** *p* = 8.27 × 10^−23^	** *p* = 1.53 × 10^−24^	** *p* = 1.54 × 10^−19^	** *p* = 6.81 × 10^−58^
Sucrose	10	** *p* = 2.66 × 10^−06^	―	** *p* = 4.51 × 10^−09^	** *p* = 3.91 × 10^−21^	** *p* = 3.71 × 10^−21^	** *p* = 1.56 × 10^−87^	** *p* = 4.59 × 10^−15^	** *p* = 2.24 × 10^−10^	** *p* = 1.36 × 10^−09^	** *p* = 7.86 × 10^−08^	** *p* = 8.91 × 10^−24^
Paclitaxel	0.1	** *p* = 1.48 × 10^−21^	** *p* = 4.51 × 10^−09^	―	** *p* = 4.01 × 10^−42^	** *p* = 1.54 × 10^−23^	** *p* = 2.24 × 10^−124^	** *p* = 4.18 × 10^−04^	** *p* = 9.88 × 10^−09^	** *p* = 3.02 × 10^−21^	** *p* = 6.33 × 10^−26^	** *p* = 5.81 × 10^−14^
	1	** *p* = 4.89 × 10^−40^	** *p* = 3.91 × 10^−21^	** *p* = 4.01 × 10^−42^	―	** *p* = 3.89 × 10^−14^	** *p* = 3.51 × 10^−48^	** *p* = 8.12 × 10^−18^	** *p* = 2.56 × 10^−23^	** *p* = 9.51 × 10^−55^	** *p* = 7.30 × 10^−41^	** *p* = 2.43 × 10^−18^
Vincristine	0.003	** *p* = 7.18 × 10^−43^	** *p* = 3.71 × 10^−21^	** *p* = 1.54 × 10^−23^	** *p* = 3.89 × 10^−14^	―	** *p* = 4.18 × 10^−50^	** *p* = 6.00 × 10^−20^	** *p* = 6.80 × 10^−26^	** *p* = 1.78 × 10^−17^	** *p* = 1.55 × 10^−04^	** *p* = 2.08 × 10^−07^
	0.03	** *p* = 1.61 × 10^−136^	** *p* = 1.56 × 10^−87^	** *p* = 2.24 × 10^−124^	** *p* = 3.51 × 10^−48^	** *p* = 4.18 × 10^−50^	―	** *p* = 4.96 × 10^−81^	** *p* = 2.18 × 10^−103^	** *p* = 1.96 × 10^−106^	** *p* = 1.19 × 10^−56^	** *p* = 5.01 × 10^−85^
Oxaliplatin	10	** *p* = 3.77 × 10^−29^	** *p* = 4.59 × 10^−15^	** *p* = 4.18 × 10^−04^	** *p* = 8.12 × 10^−18^	** *p* = 6.00 × 10^−20^	** *p* = 4.96 × 10^−81^	―	* *p* = 0.040	** *p* = 1.50 × 10^−38^	** *p* = 6.17 × 10^−32^	** *p* = 2.41 × 10^−19^
	100	** *p* = 8.27 × 10^−23^	** *p* = 2.24 × 10^−10^	** *p* = 9.88 × 10^−09^	** *p* = 2.56 × 10^−23^	** *p* = 6.80 × 10^−26^	** *p* = 2.18 × 10^−103^	* *p* = 0.040	―	** *p* = 7.09 × 10^−33^	** *p* = 6.22 × 10^−32^	** *p* = 1.64 × 10^−17^
Suramin	10	** *p* = 1.53 × 10^−24^	** *p* = 1.36 × 10^−09^	** *p* = 3.02 × 10^−21^	** *p* = 9.51 × 10^−55^	** *p* = 1.78 × 10^−17^	** *p* = 1.96 × 10^−106^	** *p* = 1.50 × 10^−38^	** *p* = 7.09 × 10^−33^	―	** *p* = 1.97 × 10^−04^	** *p* = 1.20 × 10^−06^
	100	** *p* = 1.54 × 10^−19^	** *p* = 7.86 × 10^−08^	** *p* = 6.33 × 10^−26^	** *p* = 7.30 × 10^−41^	** *p* = 1.55 × 10^−04^	** *p* = 1.19 × 10^−56^	** *p* = 6.17 × 10^−32^	** *p* = 6.22 × 10^−32^	** *p* = 1.97 × 10^−04^	―	*p* = 0.067
Bortezomib	0.01	** *p* = 6.81 × 10^−58^	** *p* = 8.91 × 10^−24^	** *p* = 5.81 × 10^−14^	** *p* = 2.43 × 10^−18^	** *p* = 2.08 × 10^−07^	** *p* = 5.01 × 10^−85^	** *p* = 2.41 × 10^−19^	** *p* = 1.64 × 10^−17^	** *p* = 1.20 × 10^−06^	*p* = 0.067	―

*: *p* < 0.05, **: *p* < 0.01.

**Table 4:** Comparison with concentrations at which toxicity was detected in previous reports.

		Toxicity Detection Concentration (µM)		Toxicity Detection (This Work)			Reference
Compound	Concentration Tested (µM)	Cell	Axon	Cell	Axon	MANOVA	
		Previous Report	Previous Report				
Paclitaxel	0.1	over 0.1	Over 0.01	◎	○	◎	[31]
	1			◎	◎	◎	
Vincristine	0.003	24 h no effect	24 h Over 0.001	○	◎	◎	[23]
	0.03	72 h 0.1		○	◎	◎	
Oxaliplatin	10	Over 10	Over 3.3	◎	○	◎	[29]
	100			◎	○	◎	
Suramin	10	48 h 300	48 h 200	○	○	◎	[33,38]
	100	8 day Over 100		○	○	◎	
Bortezomib	0.01	24 h no effect	24 h 1	○	○	◎	[23]
		72 h 0.012	72 h 0.03				

○: Over toxicity prediction line; ◎: *p* < 0.05.

## Data Availability

The data and scripts that support the findings of this study are available from the corresponding author upon reasonable request.
